# Three surgical planes identified in laparoscopic complete mesocolic excision for right-sided colon cancer

**DOI:** 10.1186/s12957-015-0758-4

**Published:** 2016-01-12

**Authors:** Da-Jian Zhu, Xiao-Wu Chen, Man-Zhao OuYang, Yan Lu

**Affiliations:** Department of Gastrointestinal Surgery, Shunde First People’s Hospital Affiliated to Southern Medical University, #1 Penglai Road, Shunde, Foshan, Guangdong Province 528300 China

**Keywords:** Colon cancer, Laparoscopic complete mesocolic excision, Surgical plane, Embryology, Imaging anatomy

## Abstract

**Background:**

Complete mesocolic excision provides a correct anatomical plane for colon cancer surgery. However, manifestation of the surgical plane during laparoscopic complete mesocolic excision versus in computed tomography images remains to be examined.

**Methods:**

Patients who underwent laparoscopic complete mesocolic excision for right-sided colon cancer underwent an abdominal computed tomography scan. The spatial relationship of the intraoperative surgical planes were examined, and then computed tomography reconstruction methods were applied. The resulting images were analyzed.

**Results:**

In 44 right-sided colon cancer patients, the surgical plane for laparoscopic complete mesocolic excision was found to be composed of three surgical planes that were identified by computed tomography imaging with cross-sectional multiplanar reconstruction, maximum intensity projection, and volume reconstruction. For the operations performed, the mean bleeding volume was 73 ± 32.3 ml and the mean number of harvested lymph nodes was 22 ± 9.7. The follow-up period ranged from 6–40 months (mean 21.2), and only two patients had distant metastases.

**Conclusions:**

The laparoscopic complete mesocolic excision surgical plane for right-sided colon cancer is composed of three surgical planes. When these surgical planes were identified, laparoscopic complete mesocolic excision was a safe and effective procedure for the resection of colon cancer.

## Background

The concept and procedure for complete mesocolic excision (CME) was initially proposed by Hohenberger et al. [1, 2] in 2009, and it provided a correct anatomical plane and surgical approach for most cases of colon cancer. During en bloc resection of the colon, the fascial space of the posterior lobe of the mesocolon is completely dissected to separate the fusion fascia between the visceral fascia and the parietal fascia up to the radix of the mesocolon. As a result, the next higher level of blood vessels that need to undergo ligaturing are revealed. The colonic vessel roots also need to be cut off in order to obtain the maximum amount of lymphoid tissue [1–4].

The fascia space [5, 6] has no major blood vessels or nerves and can be conveniently and safely separated and modified. CME follows this fascial space as a natural surgical plane by which to perform an excision. As techniques have developed and improved, laparoscopic colon cancer surgery has been able to achieve the same effect as open surgery for colon cancer [7–11]. Furthermore, a greater number of surgeons are realizing the importance of CME and the feasibility of laparoscopic complete mesocolic excision (LCME) technology is increasingly being recognized [12–14]. However, the sight line for CME is from near to far, and the visual angle is restricted by the puncture location used in laparoscopic surgery. Therefore, manifestation of the LCME surgical plane during colon cancer surgery versus visualization of the LCME surgical plane with computed tomography (CT) imaging remains to be examined.

Therefore, the aim of this study was to identify the composition and spatial relationship of the surgical planes for LCME for right-sided colon cancer, as well as the surgical planes observed by CT imaging for LCME, based on observed anatomy, CT imaging of anatomical features, and embryonic development of the gastrointestinal tract.

## Methods

### Patients

Patients who underwent LCME for right-sided colon cancer at Shunde First People’s Hospital Affiliated to Southern Medical University approved the study between January 2011 and December 2013 were enrolled in this study. Informed consent was obtained from each patient, and this study was approved by our Institutional Ethics Committee. The inclusion criteria were as follows: (1) solitary right-sided colon adenocarcinoma, (2) preoperative CT confirmation that the tumor had not broken through the colonic serosa, and (3) cardiopulmonary and hepatorenal functions were suitable for laparoscopic surgery. The exclusion criteria were as follows: (1) detection of colonic obstruction or perforation due to the tumor, (2) lung and liver metastasis, (3) patients who had undergone abdominal surgery and had severe abdominal adhesions due to intraoperative exploration and therefore were not suitable for laparoscopic surgery, and (4) the presence of concurrent malignancy.

All of the patients were examined at 3-month intervals following surgery within the first 3 years, then half-yearly in the subsequent 2 years. Chest X-rays and transabdominal ultrasonographies were performed to screen for recurrence at each follow-up session. Surveillance colonoscopy and CT scans were performed every 6 months after surgery.

### CT imaging anatomy

Each patient underwent fasting prior to being imaged in a supine position with a 128-slice CT instrument (SOMATOM Definition AS, Siemens). After observing the surgical plane of interest intraoperatively, reconstruction of the abdominal CT images was performed to display the same surgical plane. Multi-dimensional CT images of the colon were subsequently rebuilt to identify the location, composition, and spatial relationship of the LCME surgical planes for right-sided colon cancer.

### LCME

An ultra high-definition laparoscope (Stryker, USA) and Johnson & Johnson laparoscopic surgical instruments were used. Endotracheal intubation was performed under general anesthesia. The operation was carried out in a standard manner, with the patient in a supine position. The surgeon was located between the legs of the patient, the first assistant was to the right of the patient, and the assistant holding a mirror was to the left of the patient. Trocar location (5-port method): a 12-mm port for a 30° laparoscope was created below the umbilicus edge, and after establishing the pneumoperitoneum, other trocars were inserted under direct vision. The anti-McBurney point at the left lower abdomen was designated the primary operating port, while a McBurney point was the auxiliary operating port and two of the first assistant auxiliary operating ports were along the right midclavicular line.

According to the LCME procedure, the position, composition, and spatial relationships of the surgery planes needed for LCME for right-sided colon cancer cases were identified.

### Outcome measures

Intraoperative blood loss, operation time, number of lymph nodes harvested, time of first passage of flatus, postoperative hospital stay, and intraoperative and postoperative complications were observed and recorded.

CT reconstruction images were compared with each intraoperative situation.

### Statistical analysis

Statistical analyses were performed with SPSS software (SPSS Inc., USA). Kappa coefficients were used to measure agreement between CT image reconstructions and observations from laparoscopic exploratory surgery that were used to determine whether the colon cancer had invaded the surgical plane. Kappa values were used to indicate the following: strong agreement (≥0.75), good agreement (≥0.4 and <0.75), and poor agreement (<0.4). A *P* value ≤0.05 was considered statistically significant.

## Results

### Clinical data

Between January 2011 and December 2013, 47 cases at our hospital met the preoperative inclusion criteria for this study. Three cases were excluded due to tumor invasion of surrounding organs, severe abdominal adhesions, and transit to laparotomy, respectively. Thus, the remaining 44 patients were enrolled to undergo successful completion of LCME.

The follow-up period for this cohort ranged from 6–40 months (mean 21.2), and none of the patients were lost to follow-up. Postoperatively, there were no reports of anastomatic leakage, intraoperative accidental injury, or perioperative deaths. However, incision infection due to fat liquefaction (*n* = 2), intestinal adhesions and intestinal obstruction that occurred after symptomatic treatment (*n* = 1), an incisional hernia (*n* = 2), multiple liver and lung metastases detected by CT 6 months later (*n* = 1), and CT suggestive of multiple liver metastases detected 9 months later (*n* = 1) were reported (Table 1).

**Table 1:** Patient and tumor characteristics (*n* = 44)

Characteristic	*n* (%), x̄ ± s, *n*
Gender	
Male	24 (54.55 %)
Female	20 (45.45 %)
Mean age ± SD (range), year	61.16 ± 13.249 (31–83)
Tumor location	
Cecum	6 (13.64 %)
Ascending colon	24 (54.54 %)
Transverse colon (hepatic flexure)	14 (31.82 %)
Tumor type	
Massive	22 (50.00 %)
Ulcerative	17 (38.64 %)
Infiltrative	5 (11.36 %)
Maximum tumor diameter	
≥5 cm	23 (52.27 %)
<5 cm	21 (47.73 %)
TNM staging	
T_1–2_N_0_M_0_	8 (18.18 %)
T_3_N_0_M_0_	25 (56.82 %)
T_1–3_N_1–2_M_0_	11 (25.00 %)
Mean intraoperative blood loss (ml)	73 ± 32.3
Mean operation time (min)	200 ± 33.3
Lymph nodes harvested (*n*)	22 ± 9.7
Mean time of first passage of flatus (h)	74 ± 19.9
Mean hospital stay (day)	10 ± 2.2
Incision infection	2
Anastomotic leakage	0
Abdominal abscess	0
Perioperative death	0
SMV, ureteral, or duodenal injuries	0
Incisional hernia	2
Postoperative intestinal obstruction	1
Recurrence and metastasis	2

### Observed anatomical features during laparoscopy

**Fig. 1:**
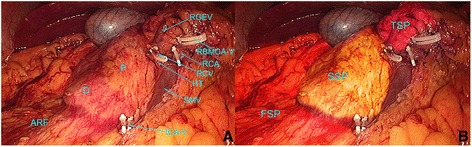
The three surgical planes in LCME for right-sided colon cancer. When the three surgical planes are superimposed, they resemble the rungs of a ladder. **a** The anatomical features involved with the LCME procedure are labeled. **b** The three surgical planes are labeled. The tissue that appears *orange* represents the right anterior renal fascia plane, which includes the FSP. The tissue that appears *yellow* includes the fusion fascia plane of the pancreatic head and the anterior layer duodenum fascia, and this represents the SSP. The tissue that appears *purple* is the space plane that is separated from the posterior lobe of the transverse mesocolon and the dorsal mesogastrium fusion fascia, and it represents the TSP. *RBMCA-V* right branch of the middle colic artery vein, *RCA* right colic artery, *HT* Henle trunk, *ICA-V* ileocolic artery vein, *ARF* anterior renal fascia

Analyses of the LCME procedures performed for right-sided colon cancer in 44 patients showed three surgical planes. When these planes were superimposed, they appeared as rungs of a ladder. The first surgical plane (FSP) included the fascia space between the posterior lobe of the ascending mesocolon and the prerenal fascia, to the right of Toldt’s space plane. The caudal boundary of the FSP was at the lower edge of the ileocolic vessels (ICV), the medial boundary was at the right edge of the superior mesenteric vein (SMV), the outer edge was the descending and lower edge of the horizontal part of the duodenum, the cranial boundary was the hepatocolic ligament, and the lateral boundary was the right paracolic sulcus. The second surgical plane (SSP) was between the posterior lobe of the ascending mesocolon and the anterior lobe of the pancreatic head and duodenum fascia. The caudal boundary of the SSP included the lower edge of the horizontal part of the duodenum, the medial boundary was the right edge of the SMV, the lateral boundary was the outer edge of the descending part of the duodenum, and the cranial boundary was a connecting line according to the radix of the middle colon vessels, the radix of the right gastroepiploic vein (RGEV), and the stomachus pyloricus (i.e., the transverse mesocolon root). The third surgical plane (TSP) was formed by the posterior lobe of the right-sided transverse mesocolon and the right-sided dorsal mesogastrium fusion fascia. The caudal and lateral TSP boundaries included the cranial boundary of the SSP, the medial boundary was the midline between the left and right branches of the middle colon vessels in the transverse mesocolon, and the cranial boundary was the gastrocolic ligament (Fig. 1a, b).

### Imaging anatomical features

**Fig. 2:**
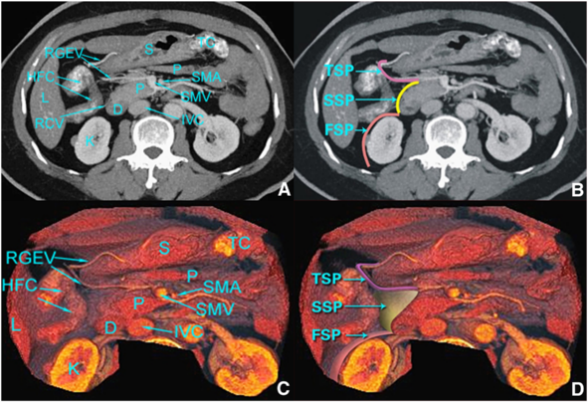
Following the administration of a dilute barium colon enema, MPR (**a**), MIP (**b**), and VR (**c**, **d**) are shown in the CT portal venous phase. The relevant anatomical features are labeled. *RGEV* right gastroepiploic vein, *FSP* first surgical plane, *HFC* hepatic flexure of colon, *SSP* second surgical plane, *L* liver, *RCV* right colic vein, *K* kidney, *TSP* third surgical plane, *S* stomach, *P* pancreas, *D* duodenum, *TC* transverse colon, *SMA* superior mesenteric artery, *SMV* superior mesenteric vein, *IVC* inferior vena cava

Surgical planes appear as linear images on CT images and are formed by two fusioned, adjacent planes. The kidney and ureter were at the back of the FSP, the SSP was in front of the anterior lobe of the pancreatic head and duodenum fascia, and the TSP was found between the posterior lobe of the right-sided transverse mesocolon and the right-sided dorsal mesogastrium fusion fascia. In the TSP, the RGEV was regarded as the central axis, the middle colic artery vein was the inner boundary, and the edges of the gastric antrum and the duodenal bulb served as the outer boundaries. The distribution of blood vessels in the three planes was also detected in the CT portal venous phase following multiplanar reconstruction (MPR), maximum intensity projection (MIP), and volume reconstruction (VR). The three surgical planes that were observed in the preoperative CT images are shown in Fig. 2.

### CT reconstruction images versus intraoperative situations: a comparative study

For the 47 cases which met the inclusion criteria for this study preoperatively, a statistical analysis was performed to compare the corresponding CT image reconstructions with the observations made during each laparoscopic exploratory surgery. In 42 cases, the colon cancer did not invade the surgical plane, while in two cases, tumor invasion of the surgical plane was observed (Table 2). However, in the latter two cases, one case showed invasion of the surgical plane in the CT imaging reconstruction and invasion was not noted in the laparoscopic surgery observations, while in the second case, invasion of the surgical plane was not detected in the CT imaging reconstruction and it was noted in the laparoscopic surgery observations. With cancer invasion of the surgical plane observed in the laparoscopic procedures considered the gold standard, the accuracy of the reconstructed CT images had a sensitivity of 93.6 % (44/47), and the false positive and false negative rates were 4.3 % (2/47) and 2.1 % (1/47), respectively. The kappa value was 0.538 (Table 2).

**Table 2:** A comparison of CT image reconstructions and laparoscopic exploratory surgery observations regarding colon cancer invasion of the surgical plane

Laparoscopic surgery observations	CT imaging reconstruction			Kappa value	*P* value
	No invasion	Invasion	Sum		
No invasion	42	2	44		
Invasion	1	2	3	0.538	0.000
Sum	43	4	47		

## Discussion

The main technical point of CME involves sharp dissection of the surgical plane to completely free the mesocolon. This also exposes the radix of mesentery, and the mesenteric vessels need to be ligated in order to obtain the lymph nodes of interest [1–4, 15–17]. In the present study, it is demonstrated that the surgical plane of LCME for right-sided colon cancer consists of three planes.

### Embryonic development and the anatomy associated with the three planes of LCME

During the embryonic period, the foregut is rotated clockwise along the central axis and the midgut is rotated counterclockwise along the superior mesenteric artery. These processes occur during different stages of embryonic development for the gastrointestinal tract, and the result is that the CME plane of the right-sided colon is not a single plane.

**Fig. 3:**
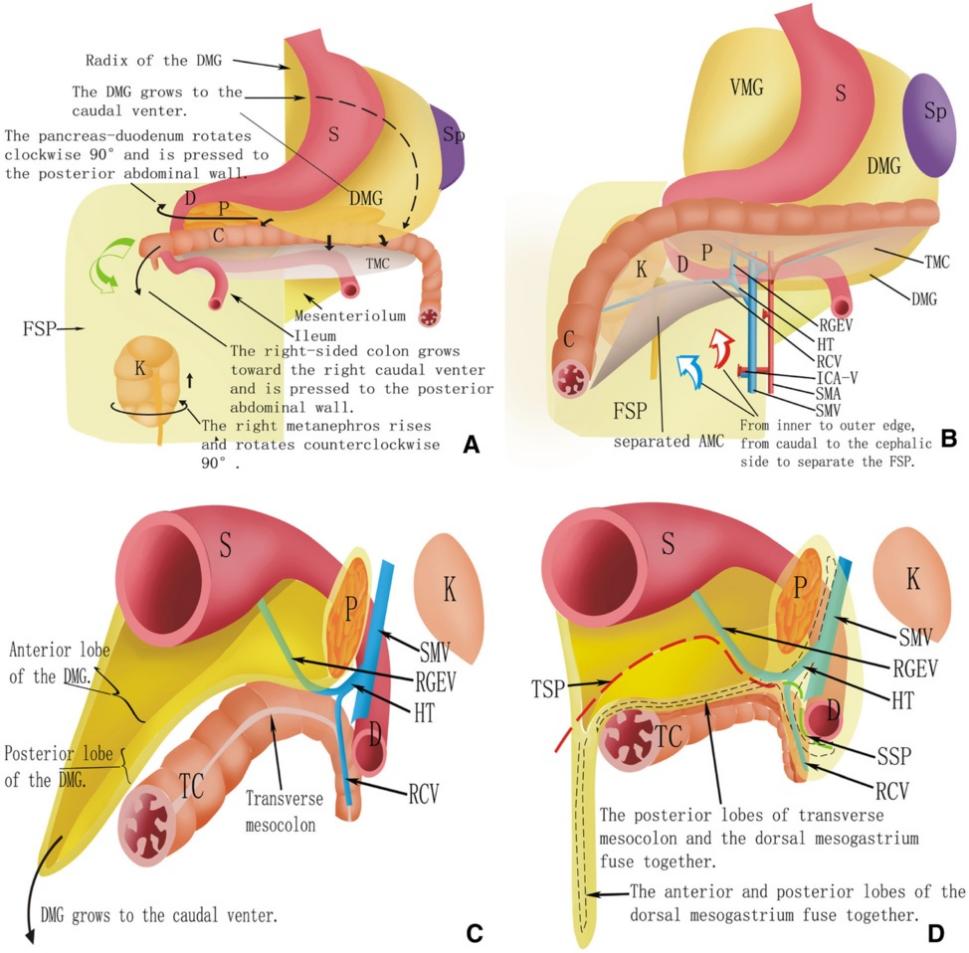
An overview of the different embryonic development periods of the gastrointestinal tract in relation to the forming process of the three surgical planes identified for LCME. *S* stomach, *Sp* spleen, *DMG* dorsal mesogastrium, *TMC* transverse mesocolon, *D* duodenum, *P* pancreas, *C* colon, *K* kidney, *ARF* anterior renal fascia, *VMG* ventral mesogastrium, *RGEV* right gastroepiploic vein, *HT* Henle trunk, *RCV* right colic vein, *ICA-V* ileocolic artery vein, *SMA* superior mesenteric artery, *SMV* superior mesenteric vein, *AMC* ascending mesocolon, *TC* transverse colon, *TSP* third surgical plane, *SSP* second surgical plane

At the second month of the embryonic period, the foregut along the central axis rotates clockwise 90°. Meanwhile, the dorsal mesogastrium (omentum majus) is convexed towards the left and starts to grow from the front of the pancreatic tail to the caudal abdomen, thereby generating the omental bursa. However, on the right side of the middle colon vessels, the anterior and posterior lobes of the dorsal mesogastrium fuse together to form the right side of the dorsal mesogastrium which is marked by the RGEV. This is the posterior boundary of the TSP (Fig. 3a).

At the fourth month, due to the development of the small intestine, the pancreas-duodenum is pressed to the posterior abdominal wall. The posterior lobe of the pancreas-duodenum fascia and the original parietal peritoneum fuse together to form Treitz’s gap with the anterior renal fascia [18]. The posterior boundary of the SSP is the anterior lobe of the pancreatic head and duodenum fascia, while the anterior renal fascia is the posterior boundary of the FSP. Taken together, these boundaries define the three surgical planes of LCME (Fig. 3a).

At the fifth month, as the small intestine continues to grow, the right-sided colon is pressed to the posterior abdominal wall (Fig. 3a). The posterior lobe of the ascending mesocolon and the original parietal peritoneum fuse into right Toldt’s fascia. The FSP includes the space between right Toldt’s fascia and the right anterior renal fascia, which is named right Toldt’s space plane (Fig. 3b). The posterior lobe of the ascending mesocolon and the anterior lobe of the pancreatic head and duodenum fascia fuse into the SSP. The dorsal mesogastrium (DMG) continues to grow to the caudal venter (Fig. 3c). The posterior lobe of the right-side transverse mesocolon and the right side of the DMG fuse together thereby forming the TSP, which is marked by the RGEV (Fig. 3d).

### A surgical path based on these three surgical planes

In this study, a caudal right surgical approach involving marking of the ICV was performed in order to adopt LCME for right-sided colon cancer (Fig. 3b). In this way, the FSP-right Toldt’s space plane is easily accessed. Upon revealing the roots of the ICV and the SMV, an incision is made on the inner edge of the ascending mesocolon on the SMV surface, with the front lobe of the pancreas-duodenum fascia used as the boundary for expanding the SSP. Then, by moving along the SMV to reveal the radix of Henle dry and the radix of the middle colonic artery and its branches, the right colic vein (RCV) and RGEV are revealed. The RCV is cut off and RGEV is used as the posterior boundary to expand the TSP to the medial. Following ligation of the radix artery and abscission of the right branch of the middle colonic artery, the TSP can be further expanded to the cephalic side (Fig. 1a, b). Finally, in the periphery dividing the greater omentum, the gastrocolic ligament, the hepatocolic ligament, and the right paracolic sulci peritoneum are incised to complete the LCME procedure. This surgical path conforms to the characteristics of laparoscopic sight from near to far and conforms to the concept of CME.

### Imaging anatomy versus the observed anatomy of the three planes of LCME during laparoscopy

In the present study, reconstructed CT images and observations from the laparoscopic exploratory surgeries performed showed good agreement, with a kappa value of 0.538 (Table 2). Thus, many insights were gained from these comparisons. For example, if the preoperative CT images showed that Toldt’s space plane was compromised by a tumor, then the operation was predicted to be very difficult. When Toldt’s space plane was found to be to the right of the FSP in the preoperative CT images (Fig. 2d), this was found to correspond with the orange shaded region in Fig. 1b. By visualizing Toldt’s line in a CT image, a surgeon can easily separate and enter the right Toldt’s space with a caudal right surgical approach marked by the ICV. Then along the SMV, by cranially separating Toldt’s line, the kidney, ureter, and reproductive vessels are in the rear of the anterior renal fascia [19], while the right-sided colon vessels are located in front of the anterior renal fascia, consistent with the CT images.

The SSP is shown in Figs. 1a and 2d, and this plane corresponds to the yellow shaded area in the intraoperative images in Fig. 1b. During surgery, if the FSP is cranially separated, this could easily be misidentified as Treitz’s space. Therefore, it is important that the CT images are consulted during surgery until finding the duodenum, and then there should be a “climbing” separation along the FSP to enter the SSP.

**Fig. 4:**
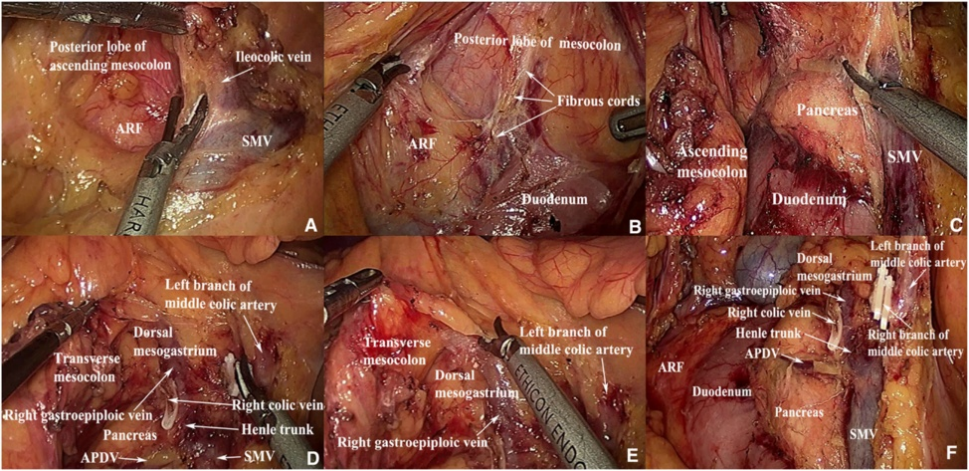
Images that represent the location of the FSP, SSP, and TSP. **a**, **b** The FSP is located between the posterior lobe of the ascending mesocolon and the ARF. Separation of the SSP is shown in (**c**). **d**, **e** The TSP is located between the transverse mesocolon and the dorsal mesogastrium. **f** The relationship between the FSP, SSP, and TSP is shown. *ARF* anterior renal fascia, *SMV* superior mesenteric vein, *APDV* anterior pancreaticoduedenal vein, *FSP* first surgical plane, *SSP* second surgical plane, *TSP* third surgical plane

The TSP is shown in Figs. 1b and 2d, and it is shaded purple in the intraoperative images in Fig. 1b. During surgery, if the SSP is followed, it is easy to cause injury to the SMV, pancreas, RGEV, duodenum, and other organs. Therefore, it remains important to pay attention to the climbing separation from the SSP into the TSP. In our experience, it helps to follow along the SMV to find the “Henle trunk” and RGEV, and then make a climbing separation along the RGEV to the TSP (Fig. 4).

### The prospective significance of preoperative CT images for LCME

For LCME, especially right-sided transverse colon cancer, it remains controversial whether the fat should be removed from around the RGEV or if the sixth group of lymph nodes in gastric cancer should be removed. We believe that if a tumor has invaded the TSP, which includes the funicular or fuzzy fat space on the TSP in CT images, then the group of lymph nodes may have been invaded by the tumor and they need to be removed. However, if a CT image shows the TSP is clear, then we recommend that the lymph nodes do not need to be removed.

As described above, preoperative CT images can display an invasion of the three surgical planes of LCME for right-sided colon cancer, and these images can be used to evaluate possible intraoperative situations. This is a key advantage for multidisciplinary teams [20, 21]. For example, preliminary results have shown that if a CT image shows that a tumor has compromised the surgical plane line, intraoperative blood loss and operation time will increase. If there are important organs around the surgical plane that are easily injured, this also increases the potential for severe complications [19]. If the preoperative CT images show that a tumor has invaded the CME surgical plane, to achieve a negative circumferential resection margin [22] (e.g., R0 removal [23]), either a deeper plane can be selected which may lead to greater damage to proximal organs and tissues from different embryonic sources, or a new adjuvant chemotherapy can be selected prior to surgery [24]. Generally, we prefer the latter option to achieve a better treatment effect.

## Conclusions

In summary, there are three surgical planes for LCME for right-sided colon cancer, and both embryonic development and anatomical observations support the presence of these planes. Moreover, we have been able to safely and effectively perform LCME by identifying these surgical planes. However, there are statistical analyses and comparisons that are missing from the present study, including changes in the R0 resection and postoperative recurrence rates. Therefore, studies are ongoing to confirm the present results and to provide more adequate experimental data for the identification and use of these three surgical planes in LCME.
